# Frailty and geriatric complications in older patients with IBD: a nationwide hospital claims database study across Japan

**DOI:** 10.1007/s40520-026-03390-8

**Published:** 2026-04-29

**Authors:** Akio Shimizu, Akira Horiuchi, Yasutake Tomata, Shintaro Togashi, Ichitaro Horiuchi, Naoharu Mori, Ryo Momosaki

**Affiliations:** 1 Department of Rehabilitation Medicine, Mie University Graduate School of Medicine, 2-174 Edobashi, Mie 514-8507 Tsu, Japan; 2 Palliative and Supportive Medicine, Graduate School of Medicine, Aichi Medical University, 1-1 Yazakokarimata, Aichi 480-1195 Nagakute, Japan; 3 Digestive Disease Center, Showa Inan General Hospital, 3230 Akaho, Nagano 399-4117 Komagane, Japan; 4 School of Nutrition and Dietetics, Faculty of Health and Social Services, Kanagawa University of Human Services, 1-10-1 Heiseicho, Kanagawa 238-0013 Yokosuka, Japan; 5 Department of Medical Data Service, TXP Medical Co. Ltd, 3-21-24 Kanda Sakumacho, 101-0025 Tokyo, Japan; 6 Department of Gastroenterology, Shinshu University Hospital, 3-1-1 Asahi, Nagano 390-0802 Matsumoto, Japan

**Keywords:** Inflammatory bowel disease, Frailty, Age, Hospitalization, Geriatric syndrome, Activities of daily living

## Abstract

**Background:**

Older patients with inflammatory bowel disease (IBD) often have multimorbidity and functional vulnerability, which increases the risk of in-hospital complications. This study examined whether frailty at admission is associated with hospital-associated geriatric complications in this population.

**Methods:**

This retrospective cohort study was conducted using a nationwide hospital-based claims database in Japan. Patients aged ≥ 60 years hospitalized with ulcerative colitis or Crohn’s disease between February 2014 and September 2025 were identified. Frailty was assessed using the Hospital Frailty Risk Score (HFRS ≥ 5). The primary outcome was hospital-associated complications in older people (HAC-OP), which is a composite of functional decline, incontinence, delirium, pressure injury, and falls/fractures. The risk ratios (RRs) and 95% confidence intervals (CIs) were estimated using modified Poisson regression.

**Results:**

Among 3,905 hospitalizations, 1,220 (31.2%) patients were classified as frail. Overall, 6.7% of hospitalizations were complicated by at least one HAC-OP, with a higher incidence in patients with frailty than in non-frail patients (10.7% vs. 4.8%). Frailty was associated with HAC-OP (adjusted RR 1.74, 95% CI 1.36–2.21), functional decline (adjusted RR 1.54, 95% CI 1.12–2.11), and delirium (adjusted RR 2.50, 95% CI 1.66–3.76). Results were robust in sensitivity analyses using multiple imputation, additional adjustment for length of stay.

**Conclusions:**

In hospitalized older patients with IBD, frailty at admission was associated with hospital-associated geriatric complications, particularly delirium and functional decline. The HFRS may be a useful tool for identifying patients at higher risk, and future studies should evaluate whether frailty-targeted interventions can reduce these complications.

**Supplementary Information:**

The online version contains supplementary material available at 10.1007/s40520-026-03390-8.

## Introduction

As the worldwide population ages, the number of older adults with inflammatory bowel disease (IBD) continues to grow [1, 2]. IBD, which includes ulcerative colitis (UC) and Crohn’s disease (CD), is a chronic, relapsing-remitting condition. The management of older patients with IBD must extend beyond controlling intestinal inflammation to address geriatric risks that can substantially influence short- and long-term outcomes.

Older patients with IBD are more prone to in-hospital complications than are younger patients [3, 4]. Multimorbidity, polypharmacy, and immunosenescence further increase the susceptibility to infection and postoperative complications [1, 4, 5]. Hospitalization itself can precipitate hospital-associated geriatric complications, such as delirium, functional decline, and new-onset incontinence, which may persist after discharge and contribute to institutionalization and mortality [6–8]. These complications represent potentially modifiable intermediate outcomes; their occurrence during hospitalization has been linked to prolonged hospitalization, institutional discharge, and increased mortality in older adults [6–8].

Frailty assessment may provide a clinically useful framework for identifying hospitalized older patients with IBD who have a heightened risk of such complications. Frailty is a multidimensional syndrome characterized by reduced physiological reserves and increased vulnerability to stressors [9]. Frailty is common in IBD, and its prevalence increases with age [10]. Frailty is associated with infection-related hospitalization, postoperative complications, readmission, and mortality [10]. These findings establish frailty as a prognostic marker of IBD. However, prior research on frailty in IBD has focused predominantly on mortality and readmission outcomes [10], whereas data on frailty and in-hospital geriatric complications in older patients with IBD remain limited. The chronic inflammatory burden and treatment patterns in older adults with IBD may further increase their vulnerability to these complications during hospitalization. Clarifying the association between frailty and HAC-OP may inform risk stratification and guide preventive strategies.

Therefore, this study examined the association between frailty status at admission and the risk of hospital-associated geriatric complications in older patients with IBD. This study also explored whether this association varied according to patient characteristics, including sex, age, and disease severity.

## Materials & methods

### Study design and participants

This retrospective cohort study utilized a large hospital-based database maintained by JMDC Inc. (Tokyo, Japan). This database integrates claims data from health insurance associations with diagnostic procedure combination (DPC) data, laboratory results, and clinical information from over 930 hospitals [11, 12]. All hospitalizations between February 2014 and September 2025 were identified. The source population comprised patients aged ≥ 60 years hospitalized with a primary diagnosis of CD or UC (ICD-10 codes K50 and K51). To maintain independence of observations and avoid potential distortion of incidence estimates due to repeated admissions within the same patient, the analysis was restricted to the first hospitalization per patient; confounding was addressed using multivariable regression. Hospitalizations resulting in in-hospital death were excluded, as the database lacks precise timing for in-hospital complications.

### Ethical considerations

This study used the DPC database provided by JMDC, Inc. (Tokyo, Japan), which contains fully anonymized administrative claims data from acute care hospitals in Japan. The Institutional Review Board of Mie University Hospital waived the requirements for ethical approval and informed consent because of the anonymized nature of the data. This study adhered to the Strengthening the Reporting of Observational Studies in Epidemiology guidelines [13] and REporting of Studies Conducted using Observational Routinely Collected Health Data guidelines [14].

### Definition of hospital-associated geriatric complications

The primary outcome was hospital-associated geriatric complications, defined according to the concept of hospital-associated complications in older people (HAC-OP) [6, 7]. HAC-OP was considered present if any of the following five geriatric syndromes occurred during the index hospitalization: (1) functional decline, defined as any decrease in the total Barthel Index (BI) [15] score from admission to discharge; (2) incontinence, defined as a decrease in BI item scores for bladder or bowel control; (3) delirium, identified by ICD-10 code F05 and/or the post-admission administration of haloperidol or other antipsychotics; (4) pressure injury, identified by ICD-10 codes recorded as post-admission complications; and (5) falls or fall-related fractures, identified by ICD-10 codes recorded as post-admission complications and/or DPC-based incident fall reports. Detailed definitions are provided in **Supplementary Table 1**. In secondary analyses, individual HAC-OP components with sufficient events were examined separately.

### Frailty assessments

Frailty was evaluated using the Hospital Frailty Risk Score (HFRS), a validated claims-based measure developed by Gilbert et al. that identifies frailty using 109 ICD-10 diagnostic codes weighted according to their association with frailty syndromes [16]. In patients with IBD, HFRS has been applied to administrative healthcare data and has shown predictive validity for adverse outcomes, including readmission and mortality [10]. The HFRS was calculated using ICD-10 diagnosis codes recorded during the 24 months preceding the index admission, consistent with the original validation study [16]. Patients were classified as frail (HFRS ≥ 5) or non-frail (HFRS < 5), consistent with previously established cut-off values [10].

### Proxy indicators for IBD severity

Disease severity was operationalized using treatment intensity as a proxy for disease activity, consistent with the 2020 Evidence-based Clinical Practice Guidelines for IBD in Japan [2]. As detailed clinical indices were unavailable in the claims data, severity was classified into three grades (severe, moderate to severe, and mild-to-moderate) based on the most intensive therapy received during index hospitalization.

Patients with UC were classified as having severe disease if they required surgical intervention or received a combination of calcineurin inhibitors and systemic corticosteroids. Moderate to severe disease was defined as the use of at least one intensive medical therapy, including systemic corticosteroids, calcineurin inhibitors, biologics, Janus kinase inhibitors, or granulocyte and monocyte adsorption apheresis (GMA) in patients who did not meet the criteria for severe disease. Mild-to-moderate disease comprises patients managed without intensive therapies, such as those treated with 5-aminosalicylic acid (5-ASA), rectal corticosteroids, or immunomodulators.

Patients with CD were classified as having severe disease if they required intestinal surgical intervention or received a combination of total parenteral nutrition (TPN) and systemic corticosteroids. Moderate to severe disease was defined as the use of at least one intensive therapy (systemic corticosteroids, biologics, Janus kinase inhibitors, GMA, or TPN) in the absence of criteria for severe disease. Mild-to-moderate disease was defined as disease managed without intensive therapy (e.g., 5-ASA, enteral nutrition, or immunomodulators).

Immunomodulators (azathioprine or 6-mercaptopurine) are categorized as treatments for mild-to-moderate disease because they are primarily used for maintenance rather than acute induction in hospitalized patients. Hospitalizations involving dialysis were excluded from the intensive GMA therapy count. The detailed definitions and treatment combinations for each severity category are summarized in **Supplementary Table 2**. The specific medications and procedure codes used are listed in **Supplementary Table 3**.

### Covariates

Demographic and clinical characteristics included age, sex, body mass index (BMI), Charlson Comorbidity Index (CCI) [17], Brinkman index (pack-years), and Barthel Index (BI) at admission [15]. Disease-specific factors included the IBD category (UC or CD) and the time from first recorded IBD diagnosis to index admission, defined as the number of days between the earliest recorded outpatient visit with an IBD-related diagnosis code at the participating hospital and the index admission date. As the database was derived from hospital-based administrative claims, the exact date of disease onset at other facilities could not be ascertained. Healthcare system factors included hospital bed size and year of admission.

### Statistical analysis

Baseline characteristics were summarized according to the frailty status at admission. Categorical variables are presented as counts and percentages, and continuous variables as means with standard deviations or medians with interquartile ranges, as appropriate.

In the primary analysis, the development of at least one HAC-OP during the index hospitalization was modeled as a binary outcome using an analytic cohort restricted to hospitalizations with a non-missing BI at both admission and discharge. In secondary analyses, separate outcome models were constructed for individual HAC-OP components with sufficient events. The risk ratios (RRs) and 95% confidence intervals (CIs) for the association between frailty status and HAC-OP were estimated using modified Poisson regression with robust variance estimation. Frailty was modeled as a categorical exposure, with the non-frail group as the reference category. The multivariable models were adjusted for age (continuous), sex, BMI (< 18.5, 18.5–<25, ≥ 25 kg/m², missing), CCI score (continuous), Brinkman index (0, 1–399, ≥ 400, missing), BI at admission (continuous), disease category (UC or CD), IBD severity (mild to moderate, moderate to severe, or severe), time from first recorded IBD-related diagnosis code to index admission (continuous), hospital bed size (< 200 or ≥ 200 beds), and year of admission (2014–2015, 2016–2017, 2018–2019, 2020–2021, 2022–2023, 2024–2025). To assess potential effect modification of the association between frailty and HAC-OP, multiplicative interaction terms were added between frailty status and each of the following variables: sex (male vs. female), age group (< 75 vs. ≥75 years), and IBD severity (mild to moderate vs. moderate to severe or severe). Statistical interactions were evaluated using Wald tests for interaction terms.

As a sensitivity analysis, the primary analysis was repeated using multiple imputations by chained equations (MICE) to handle missing data in the covariates and BI scores. It was assumed that these data were missing at random conditional on the observed information. Twenty imputed datasets were generated using the mouse package in R by applying the random forest imputation method. Estimates from the imputed datasets were combined using Rubin’s rule to obtain pooled RRs and 95% CIs.

An additional analysis was conducted: (1) adjusting for length of hospital stay (LOS) as a covariate, given that LOS may act as a mediator on the causal pathway from frailty to HAC-OP and that LOS-adjusted estimates should be interpreted as direct effects.

All statistical tests were two-sided, and P values < 0.05 were considered statistically significant. All analyses were performed using R (version 4.4.2; R Foundation for Statistical Computing, Vienna, Austria).

## Results

**Fig. 1:**
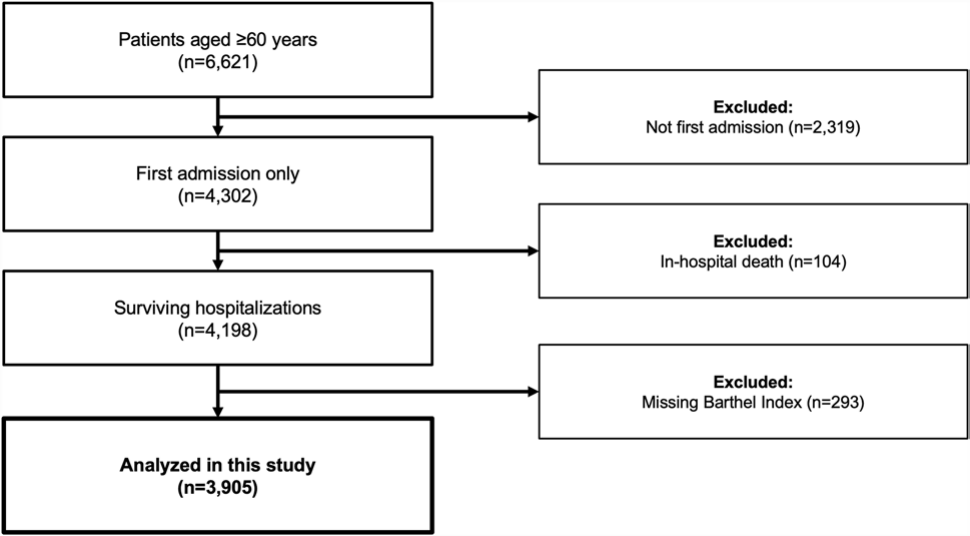
Flowchart of patient selection

During the study period, 6,621 hospitalizations for patients aged ≥ 60 years met the initial eligibility criteria. After restricting the study to the first hospitalization per patient (*n* = 4,302) and excluding 104 (2.4%) cases of in-hospital death, 4,198 hospitalizations were included in the main cohort (Fig. 1). The in-hospital mortality was substantially higher in patients with frailty compared to non-frail patients (5.3% vs. 1.0%). The analytical cohort for the primary analysis comprised 3,905 hospitalizations with non-missing BI scores at both admission and discharge.

Based on the HFRS, 1,220 patients (31.2%) were classified as frail (Table 1). Patients with frailty were older (median [IQR], 73 [66–79] vs. 69 [64–76] years) and had lower admission BI scores than the non-frail patients (median [IQR], 100 [85–100] vs. 100 [100–100]). Patients with frailty were more likely to have CD (23.4% vs. 17.8%) and higher CCI scores. Regarding treatment during hospitalization, patients with frailty were less likely to receive systemic corticosteroids (29.3% vs. 34.0%) and more likely to receive dietary provision as the initial route of nutritional intake (62.9% vs. 57.7%) (**Supplementary Table 4**).

**Table 1:** Baseline characteristics by frailty status

Variable	Overall	Frail	Non-frail
n	3,905	1,220	2,685
Age, years, median [IQR]	70 [64–77]	73 [66–79]	69 [64–76]
Male sex, n (%)	2,256 (57.8)	680 (55.7)	1,576 (58.7)
Body mass index category, n (%)			
<18.5	497 (12.7)	170 (13.9)	327 (12.2)
18.5–<25	2,265 (58.0)	690 (56.6)	1,575 (58.7)
≥25	564 (14.4)	149 (12.2)	415 (15.5)
Missing	579 (14.8)	211 (17.3)	368 (13.7)
Brinkman index category, n (%)			
0	2,231 (57.1)	711 (58.3)	1,520 (56.6)
1–399	290 (7.4)	91 (7.5)	199 (7.4)
≥400	821 (21.0)	246 (20.2)	575 (21.4)
Missing	563 (14.4)	172 (14.1)	391 (14.6)
Crohn’s disease, n (%)	764 (19.6)	286 (23.4)	478 (17.8)
Disease severity, n (%)			
Mild to Moderate	2,233 (57.2)	748 (61.3)	1,485 (55.3)
Moderate to Severe	1,420 (36.4)	410 (33.6)	1,010 (37.6)
Severe	252 (6.5)	62 (5.1)	190 (7.1)
Time from first recorded IBD diagnosis to index admission, days, median [IQR]	0 [0–9]	0 [0–289]	0 [0–3]
Charlson Comorbidity Index, median [IQR]	0 [0–1]	0 [0–1]	0 [0–1]
Barthel Index at admission, median [IQR]	100 [100–100]	100 [85–100]	100 [100–100]
Hospital bed capacity ≥ 200, n (%)	3,415 (87.5)	1,087 (89.1)	2,328 (86.7)
Year of admission, n (%)			
2014–2015	155 (4.0)	31 (2.5)	124 (4.6)
2016–2017	278 (7.1)	53 (4.3)	225 (8.4)
2018–2019	864 (22.1)	219 (18.0)	645 (24.0)
2020–2021	929 (23.8)	290 (23.8)	639 (23.8)
2022–2023	922 (23.6)	323 (26.5)	599 (22.3)
2024–2025	757 (19.4)	304 (24.9)	453 (16.9)

Frailty was defined as HFRS ≥ 5

Abbreviations: IQR, interquartile range; IBD, inflammatory bowel disease; HFRS, Hospital Frailty Risk Score

**Fig. 2:**
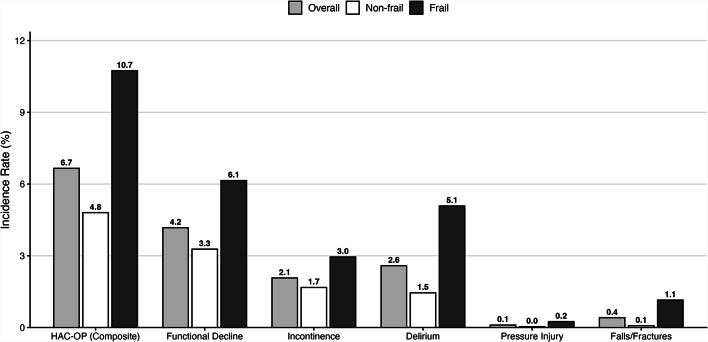
Incidence of HAC-OP components by frailty status. HAC-OP, hospital-associated complications of older people

Overall, 6.7% of the hospitalizations were complicated by at least one HAC-OP. The incidence was approximately twice as high in patients with frailty as in non-frail patients (10.7% vs. 4.8%) (Fig. 2). Specifically, functional decline occurred in 6.1% of patients with frailty and 3.3% of the non-frail patients. Similar patterns were observed in delirium (5.1% vs. 1.5%) and incontinence (3.0% vs. 1.7%).

**Fig. 3:**
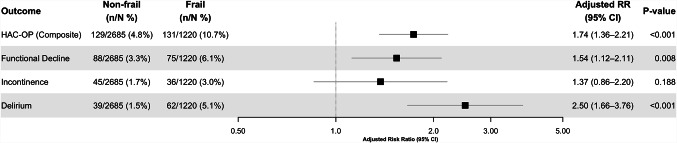
Adjusted risk ratios for HAC-OP according to frailty status. Models are adjusted for age (continuous), sex (male vs. female), body mass index (< 18.5, 18.5–<25, or ≥ 25 kg/m², missing), Charlson Comorbidity Index (continuous), Brinkman index (0, 1–399, or ≥ 400, missing), Barthel Index at admission (continuous), disease type (ulcerative colitis vs. Crohn’s disease), disease severity (mild to moderate, moderate to severe, or severe), time from first recorded inflammatory bowel disease-related diagnosis code to index admission (continuous), hospital bed size (< 200 vs. ≥200 beds), and year of admission (2014–2015, 2016–2017, 2018–2019, 2020–2021, 2022–2023, or 2024–2025). HAC-OP, hospital-associated complications in older people; RR, risk ratio; CI, confidence interval

In multivariable models, frailty was associated with a higher risk of HAC-OP (adjusted RR 1.74, 95% CI 1.36–2.21) (Fig. 3). Frailty was also associated with functional decline (adjusted RR 1.54, 95% CI 1.12–2.11) and delirium (adjusted RR 2.50, 95% CI 1.66–3.76). For incontinence, the point estimate suggested a positive association; however, the CI was wide and included null (adjusted RR 1.37, 95% CI 0.86–2.20).

**Fig. 4:**
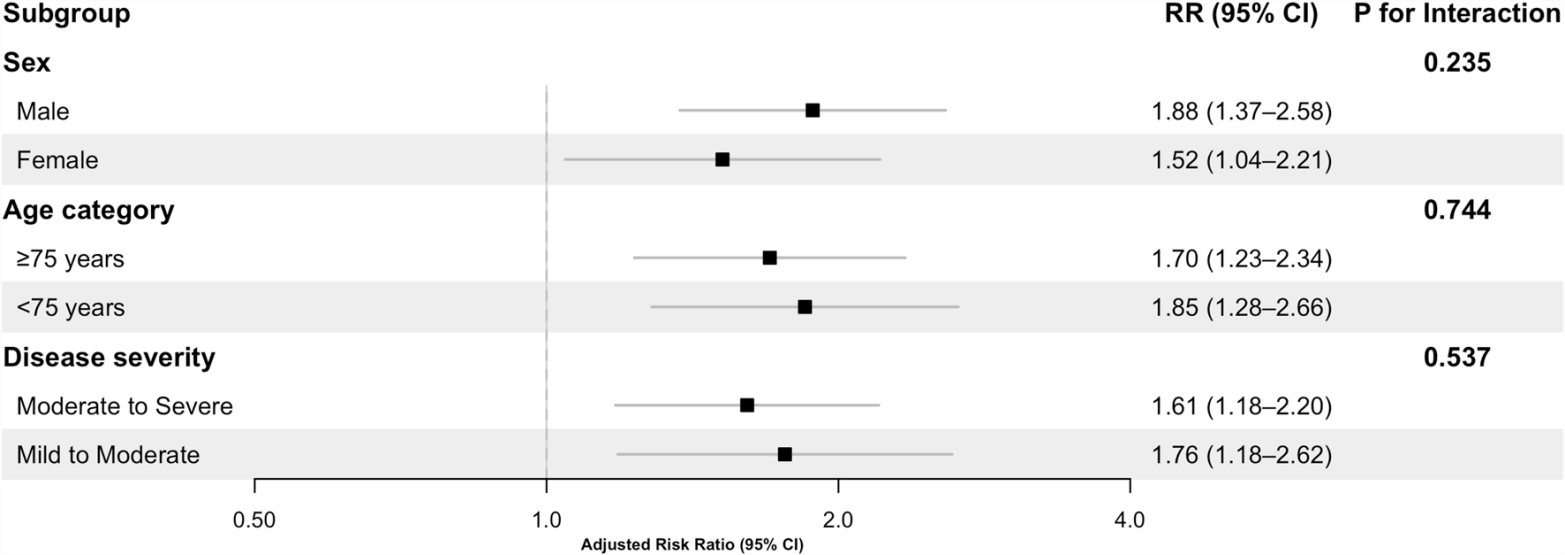
Interaction of frailty with sex, age, and IBD severity for HAC-OP. Estimates were derived from modified Poisson regression models with robust variance, including the main effects of frailty, each subgroup variable, and their product terms. Models are adjusted for age (continuous), sex (male vs. female), body mass index (< 18.5, 18.5–<25, or ≥ 25 kg/m², missing), Charlson Comorbidity Index (continuous), Brinkman index (0, 1–399, or ≥ 400, missing), Barthel Index at admission (continuous), disease type (ulcerative colitis vs. Crohn’s disease), disease severity (mild to moderate, moderate to severe, or severe), time from the first recorded IBD diagnosis to admission (continuous), hospital bed size (< 200 vs. ≥200 beds), and year of admission (2014–2015, 2016–2017, 2018–2019, 2020–2021, 2022–2023, or 2024–2025). P-values correspond to Wald tests for interactions, and no statistically significant interactions were observed. IBD, inflammatory bowel disease; RR, risk ratio; CI, confidence interval; HAC-OP, hospital-associated complications in older people

The association between frailty and HAC-OP was examined across patient subgroups (Fig. 4). The association was consistent across subgroups defined by sex (P for interaction = 0.235), age ≥ 75 years (P for interaction = 0.744), and disease severity (P for interaction = 0.537).

In the sensitivity analysis using multiple imputations (*n* = 4,198; 31.7% frail), patient characteristics were similar to those of the analytic cohort (**Supplementary Table 5**). Frailty remained associated with HAC-OP (adjusted RR 1.71, 95% CI 1.34–2.18), consistent with the primary analysis (**Supplementary Fig. 1**).

In the additional analysis adjusting for LOS, the association between frailty and HAC-OP remained robust (adjusted RR 1.63, 95% CI 1.28–2.08). Similar results were observed for functional decline (adjusted RR 1.43, 95% CI 1.05–1.96) and delirium (adjusted RR 2.30, 95% CI 1.51–3.50) (**Supplementary Fig. 2**).

## Discussion

In this nationwide study of hospitalized older patients with IBD, frailty on admission was associated with a higher risk of hospital-associated geriatric complications. Among the individual components, this association was observed for delirium and functional decline, whereas for incontinence, the point estimate suggested a positive association; however, the estimate was imprecise and did not reach statistical significance.

In this cohort of hospitalized older patients with IBD, 31.2% were classified as frail (HFRS ≥ 5). A pooled analysis of six studies reporting frailty prevalence by age found that 33% (95% CI 15.1–57.8%) of patients aged ≥ 60 or ≥ 65 years were frail [10]. The estimate falls within this range, although the wide CI reflects heterogeneity across studies and differences in frailty instruments, including administrative data-based measures, such as the HFRS. Previous studies have demonstrated that frailty in IBD is associated with higher risks of mortality, unplanned readmission, and serious medical complications [10, 18–21]. These findings extend this evidence by demonstrating that frailty is associated with hospital-related geriatric complications.

The association between frailty and HAC-OP can be explained by several mechanisms. Patients with frailty have diminished physiological reserves, making them more susceptible to stressors such as acute illness, hospitalization-related immobility, and medication effects [9]. IBD-related factors may contribute to delirium, potentially through brain-gut axis involvement, malnutrition, and aberrant cytokine production. Corticosteroid exposure, which is common in older patients with IBD, may further increase susceptibility to delirium [22]. Functional decline was also associated with frailty. Because most patients were fully independent at admission, hospitalization itself may have contributed to an acute decline in individuals with frailty, potentially exacerbated by inflammatory burden, malnutrition, and fasting. The point estimate for incontinence was positive but imprecise, which may reflect limited events, underrecording, or a stronger influence of baseline function and/or acute illness severity than frailty itself [23].

LOS may lie on the causal pathway from frailty to HAC-OP; therefore, adjusting for LOS could introduce overadjustment bias. Nevertheless, the associations remained robust after LOS adjustment, suggesting that the observed relationship is not solely explained by differences in hospitalization duration.

HAC-OP can be considered a modifiable intermediate outcome: baseline frailty and comorbidities, together with in-hospital care processes, influence HAC-OP occurrence, which in turn is associated with prolonged hospitalization, institutional discharge, and long-term mortality [6–8]. The present study quantifies the association between frailty and HAC-OP, suggesting that frailty assessment at admission may help identify patients who would benefit from targeted preventive strategies.

This study had several strengths, including the use of a large nationwide database, a validated frailty measure (HFRS), and the assessment of multiple HAC-OP components with sensitivity analyses, yielding consistent results.

The association between frailty and HAC-OP was consistent across the subgroups defined by sex, age, and disease severity, suggesting that frailty is a relevant risk factor regardless of these characteristics. However, given the exploratory nature of these analyses and the limited statistical power to detect modest interactions, these findings should be interpreted with caution.

This study had several limitations. First, HAC-OP components are underrecorded in administrative data, likely attenuating the observed associations. Despite using both ICD-10 codes and DPC-based incident fall reports for falls ascertainment, underascertainment may persist. Second, disease severity was assessed indirectly using treatment intensity and may lead to misclassification; unmeasured disease activity cannot be excluded. Third, excluding in-hospital deaths may introduce collider stratification bias, as both frailty and HAC-OP may influence death risk, potentially leading to underestimation of the association between frailty and HAC-OP. These findings may not be generalizable to other populations or healthcare systems. Fourth, the HFRS was developed using NHS data from England [10]; formal validation in Japanese DPC data has not been performed. Although several studies using Japanese DPC data have reported that the HFRS predicts adverse outcomes [24–26], its applicability in the DPC setting still requires more detailed evaluation. Fifth, patients with fewer than 24 months of data may have had incomplete ascertainment of comorbidities used to calculate the HFRS, potentially leading to misclassification as non-frail. Finally, this study did not evaluate preventive interventions in patients identified as frail. Future prospective studies should examine whether standardized, HFRS-triggered geriatric interventions (e.g., delirium prevention bundles, early rehabilitation, and nutritional strategies) can reduce the incidence of HAC-OP in older patients with IBD, and how frailty assessment can be integrated into clinical pathways or guidelines for this population.

## Conclusions

In this hospital-based cohort of older patients with IBD, frailty at admission was associated with hospital-associated geriatric complications, particularly delirium and functional decline. The HFRS may serve as a useful tool for identifying older patients with IBD at higher risk of in-hospital geriatric complications. Future prospective studies are warranted to determine whether frailty-targeted preventive strategies can reduce the incidence of these complications.

## Supplementary Information

Below is the link to the electronic supplementary material.

Supplementary Material 1

## Data Availability

The data used in this study are not publicly available due to privacy regulations and the protection of personal information.
