# Supplementary material for: Frailty and geriatric complications in older patients with IBD: a nationwide hospital claims database study across Japan
Source: Aging Clin Exp Res. 2026 Apr 29;38(1):154. doi: 10.1007/s40520-026-03390-8 (PMC13279736; doi:10.1007/s40520-026-03390-8)
Supplement: Supplementary file 1 — Supplementary Material 1 [file 40520_2026_3390_MOESM1_ESM.docx]

**Frailty and geriatric complications in older patients with IBD: A nationwide hospital claims database study across Japan**

**Supplementary Table 1.** Definitions of HAC-OP components in claims data

| HAC-OP components | ICD-10 / claims-based definition |
| --- | --- |
| Delirium | F05 and/or the post-admission administration of haloperidol or other antipsychotics |
| Pressure injury | L89 |
| Fall/Fracture | W01, W05, W06, W07, W08, W10, W18, W19, S02, S12, S22, S32, S42, S52, S62, S72, S82, S92; DPC-based incident fall reports |

All outcomes were defined as post-admission complications (i.e., not present on admission).

**Abbreviations:** HAC-OP, hospital-associated complications of older people; ICD-10, International Classification of Diseases, 10th Revision

**Supplementary Table 2.** Definitions of inflammatory bowel disease severity based on treatment intensity

| **Severity Category** | **Ulcerative Colitis** | **Crohn's Disease** |
| --- | --- | --- |
| **Severe** | Patients meeting at least one of the following:  • Requirement for urgent surgery  • Use of calcineurin inhibitors (cyclosporine or tacrolimus) combined with  systemic corticosteroids | Patients meeting at least one of the following:  • Requirement for urgent surgery  • Use of total parenteral nutrition combined with systemic corticosteroids |
| **Moderate to Severe** | Patients not meeting criteria for Severe disease but treated with at least one of the following intensive therapies:  • Systemic corticosteroids  • Calcineurin inhibitors  • Biologics (infliximab, adalimumab, golimumab, ustekinumab, vedolizumab)  • Janus kinase inhibitors  • Granulocyte and monocyte adsorption apheresis | Patients not meeting criteria for Severe disease but treated with at least one of the following intensive therapies:  • Systemic corticosteroids  • Biologics (infliximab, adalimumab, golimumab, ustekinumab, vedolizumab, risankizumab)  • Janus kinase inhibitors  • Granulocyte and monocyte adsorption apheresis  • Total parenteral nutrition |
| **Mild to Moderate** | Patients managed without the above intensive therapies. Treatment may include:  • 5-aminosalicylic acid  • Rectal corticosteroids  • Immunomodulators (azathioprine, 6-mercaptopurine) | Patients managed without the above intensive therapies. Treatment may include:  • 5-aminosalicylic acid  • Enteral nutrition  • Immunomodulators (azathioprine, 6-mercaptopurine) |

**Supplementary Table 3.** Medication and surgical codes used for inflammatory bowel disease severity assessment

| Medication or surgical name | Medication or surgical code |
| --- | --- |
| **Systemic or rectal corticosteroid** | 610408661, 610422253, 610431117, 610454071, 612450051, 612450070, 612450096, 612450118, 612450121, 620000125, 620000694, 620000695, 620000696, 620000697, 620000698, 620001310, 620001894, 620001895, 620001896, 620002208, 620002209, 620002513, 620002613, 620002614, 620002615, 620002616, 620002617, 620003272, 620003829, 620003830, 620003831, 620003832, 620004294, 620004387, 620004578, 620004620, 620004660, 620004661, 620005125, 620005126, 620005133, 620005134, 620005162, 620005163, 620005848, 620006161, 620006162, 620006275, 620006276, 620006613, 620006614, 620006903, 620006985, 620006986, 620007078, 620007332, 620007333, 620007334, 620007335, 620007336, 620007356, 620007357, 620007358, 620007359, 620007381, 620007382, 620008220, 620008651, 620008816, 620008817, 620008818, 620008819, 620519503, 620519603, 620521302, 620521501, 620525001, 620525101, 620525201, 620525301, 620525401, 620525601, 620525701, 620525801, 620525901, 620527133, 620527205, 620528103, 620528502, 620528505, 620528901, 620528905, 620529204, 620530402, 620530502, 621559301, 621977200, 621997701, 622100401, 622100501, 622329000, 622329100, 622329500, 622329600, 622329700, 622359901, 622526001, 640454024, 640454025, 640454026, 642450080, 642450081, 642450087, 642450115, 642450116, 642450117, 642450139, 642450140, 642450141, 642450142, 642450169, 642450170, 642450171, 620009010, 620009011, 660463030, 662450002, 662450003, 622564901 |
| **5-aminosalicylic acid** | 620008106, 620008107, 620008510, 620008509, 621867201, 620009440, 620008514, 620008511, 620506801, 621969902, 620009441, 621965201, 622026201, 622011801, 622234801, 622165801, 622065002, 622053401, 622173401, 622129401, 622167101, 622129501, 622458101, 622442701, 622537801, 622590701, 622517001, 622563401, 621890601, 622109402, 622416402, 621884303, 622924101, 622818901, 622102903, 622924201, 660470002, 620004897, 620008624, 620008997, 620009119, 622023001, 622173901, 622173801, 622039002, 621442905, 621442802, 622103602, 622161402, 622746900, 621133002, 622746800, 622747000, 621443003, 622833802,  610463004 |
| **Immunosuppressants** |  |
| Cyclosporine | 620882601, 621326201, 621743306, 622056201, 621743406, 622043804, 620894001, 622056401, 622056301, 621674801, 621677601, 621677701, 621738001, 621674701, 621732201, 622741800, 622741900, 622742000, 621483705, 622879101, 622879301, 621637804, 621483605, 621685604, 622879201, 610443019, 610443020, 613990086, 613990085, 610443018 |
| Tacrolimus | 620008437, 620008439, 620008438, 622181801, 622166001, 622047401, 622145501, 622146301, 622270501, 622438701, 622270701, 622281301, 622438001, 622280901, 622270601, 622281201, 622370101, 622437901, 622438601, 622438101, 622281001, 622281101, 622384303, 622370201, 622438801, 622590901, 622384603, 622384403, 622580901, 622384503, 622370001, 622384703, 622232202, 622232303, 622232403, 610409342, 610451010, 610443059, 613990096, 610451009, 643990141, 660432014, 620000444 |
| **Biologics** |  |
| Infliximab | 622378801, 622596901, 622577001, 622651501, 622590801, 640462006 |
| Adalimumab | 620006808, 622093601, 622509701, 622509801, 622609001, 622608801, 622608901, 629917701, 629911401, 629913701, 629925301, 629913601, 629911301, 629917501, 629911201, 629917601, 629925101, 629925201, 629925001 |
| Golimumab | 622070002, 622675801 |
| Ustekinumab | 622046501, 622554701, 629930501 |
| Vedolizumab | 622646301, 629923601, 629923501 1 |
| **JAK inhibitors** |  |
| Tofacitinib | 622242601 |
| Filgotinib | 622070002, 622675801 |
| **Immunomodulators** |  |
| Azathioprine | 613990075, 620004279, 620006560 |
| 6-Mercaptopurine | 620008778 |
| **Granulocytapheresis** | 140039210 |
| **Colorectal surgery** |  |
| Ulcerative colitis | 150325210, 150337810, 150297510, 150277810, 150337710, 150181710, 150181810, 150181910, 150363810 |
| Crohn's disease | 150325210, 150337810, 150297510, 150277810, 150337710, 150181710, 150181810, 150181910, 150363810, 150184710, 150181210 |
| Endoscopic balloon duration | 150293310 |

Medication codes refer to Japanese National Health Insurance reimbursement receipt drug codes, and the surgical/procedural codes are Japanese procedure billing codes from the national fee schedule, both of which are used for claims and case-mix classification in the Diagnosis Procedure Combination system.

**Supplementary Table 4.** Inflammatory bowel disease treatment characteristics by frailty status

| Variable | Overall | Frail | Non-frail |
| --- | --- | --- | --- |
| n | 3,905 | 1,220 | 2,685 |
| 5-aminosalicylic acid, n (%) | 1,745 (44.7) | 542 (44.4) | 1,203 (44.8) |
| Corticosteroids, n (%) |  |  |  |
| Systemic | 1,270 (32.5) | 358 (29.3) | 912 (34.0) |
| Rectal | 285 (7.3) | 88 (7.2) | 197 (7.3) |
| Immunosuppressants, n (%) |  |  |  |
| Cyclosporine | 17 (0.4) | 3 (0.2) | 14 (0.5) |
| Tacrolimus | 75 (1.9) | 13 (1.1) | 62 (2.3) |
| Immunomodulators, n (%) | 310 (7.9) | 100 (8.2) | 210 (7.8) |
| Biologics, n (%) |  |  |  |
| Infliximab | 93 (2.4) | 28 (2.3) | 65 (2.4) |
| Adalimumab | 71 (1.8) | 19 (1.6) | 52 (1.9) |
| Golimumab | 13 (0.3) | 6 (0.5) | 7 (0.3) |
| Ustekinumab | 33 (0.8) | 10 (0.8) | 23 (0.9) |
| Vedolizumab | 92 (2.4) | 29 (2.4) | 63 (2.3) |
| Risankizumab | 5 (0.1) | 2 (0.2) | 3 (0.1) |
| JAK inhibitors, n (%) | 50 (1.3) | 19 (1.6) | 31 (1.2) |
| Granulocyte and monocyte adsorptive apheresis, n (%) | 288 (7.4) | 74 (6.1) | 214 (8.0) |
| Urgent surgery, n (%) | 188 (4.8) | 49 (4.0) | 139 (5.2) |
| Initial route of nutritional intake, n (%) |  |  |  |
| Total parenteral nutrition | 246 (6.3) | 60 (4.9) | 186 (6.9) |
| Enteral nutrition | 278 (7.1) | 88 (7.2) | 190 (7.1) |
| Dietary provision | 2,316 (59.3) | 767 (62.9) | 1,549 (57.7) |

**Abbreviation:** JAK, Janus kinase.

**Supplementary Table 5.** Patient characteristics by frailty status: pooled estimates from multiple imputation

| **Variable** | **Overall** | **Frail** | **Non-frail** |
| --- | --- | --- | --- |
| n | 4,198 | 1,330 | 2,868 |
| Age, years, median [IQR] | 71 [64–77] | 73 [66–79] | 70 [64–76] |
| Male sex, n (%) | 2426 (57.8) | 744 (55.9) | 1682 (58.6) |
| Body mass index category, n (%) |  |  |  |
| <18.5 | 646 (15.4) | 231 (17.4) | 415 (14.5) |
| 18.5–<25 | 2844 (67.8) | 898 (67.5) | 1947 (67.9) |
| ≥25 | 708 (16.9) | 202 (15.2) | 506 (17.7) |
| Brinkman index category, n (%) |  |  |  |
| 0 | 2777 (66.2) | 901 (67.7) | 1877 (65.4) |
| 1–399 | 372 (8.8) | 115 (8.7) | 256 (8.9) |
| ≥400 | 1049 (25.0) | 314 (23.6) | 735 (25.6) |
| Crohn's disease, n (%) | 836 (19.9) | 316 (23.8) | 520 (18.1) |
| Disease severity, n (%) |  |  |  |
| Mild to Moderate | 2,402 (57.2) | 810 (60.9) | 1,592 (55.5) |
| Moderate to Severe | 1,518 (36.2) | 451 (33.9) | 1,067 (37.2) |
| Severe | 278 (6.6) | 69 (5.2) | 209 (7.3) |
| Time from first recorded IBD diagnosis to index admission, days, median [IQR] | 0 [0–10] | 0 [0–294] | 0 [0–2] |
| Charlson Comorbidity Index, median [IQR] | 0 [0–1] | 0 [0–1] | 0 [0–1] |
| Barthel Index at admission, median [IQR] | 100 [100–100] | 100 [80–100] | 100 [100–100] |
| Hospital bed capacity ≥200, n (%) | 3,681 (87.7) | 1,184 (89.0) | 2,497 (87.1) |
| Year of admission, n (%) |  |  |  |
| 2014–2015 | 179 (4.3) | 34 (2.6) | 145 (5.1) |
| 2016–2017 | 294 (7.0) | 63 (4.7) | 231 (8.1) |
| 2018–2019 | 928 (22.1) | 241 (18.1) | 687 (24.0) |
| 2020–2021 | 1,020 (24.3) | 326 (24.5) | 694 (24.2) |
| 2022–2023 | 979 (23.3) | 344 (25.9) | 635 (22.1) |
| 2024–2025 | 798 (19.0) | 322 (24.2) | 476 (16.6) |

Data are presented as median [interquartile range] or n (%). Values represent pooled estimates across 20 multiply imputed datasets. P-values were calculated using Wilcoxon rank-sum test for continuous variables and chi-square test (or Fisher's exact test when expected cell counts <5) for categorical variables; reported P-values are median values across imputed datasets.

**Abbreviations:** BMI, body mass index; IQR, interquartile range; IBD, inflammatory bowel disease.

**Supplementary Figure 1.** Adjusted risk ratios for HAC-OP according to frailty status: sensitivity analysis using multiple imputation


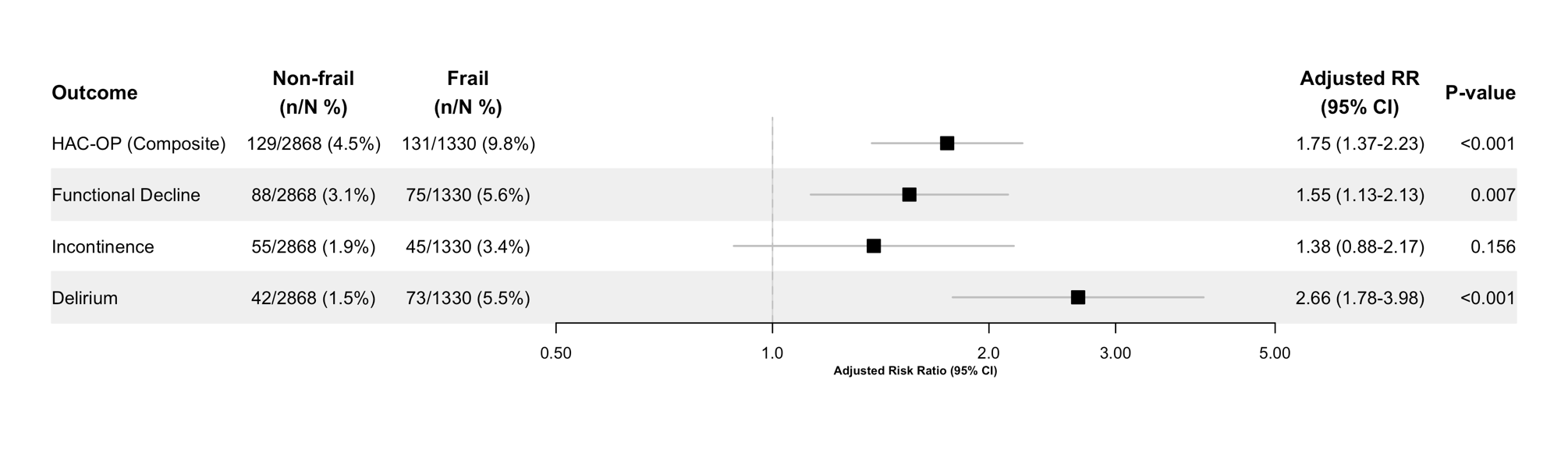


Missing values were imputed using multiple imputation with chained equations. Models are adjusted for age (continuous), sex (male vs. female), body mass index (<18.5, 18.5–<25, or ≥25 kg/m²), Charlson Comorbidity Index (continuous), Brinkman index (0, 1–399, or ≥400, Barthel Index at admission (continuous), disease type (ulcerative colitis vs. Crohn's disease), disease severity (mild to moderate, moderate to severe, or severe), time from first recorded inflammatory bowel disease-related diagnosis code to index admission (continuous), hospital bed size (<200 vs. ≥200 beds), and year of admission (2014–2015, 2016–2017, 2018–2019, 2020–2021, 2022–2023, or 2024–2025).

**Abbreviations:** HAC-OP, hospital-associated complications of older people; RR, risk ratio; CI, confidence interval.

**Supplementary Figure 2.** Adjusted risk ratios for HAC-OP according to frailty status: sensitivity analysis with additional adjustment for length of hospital stay


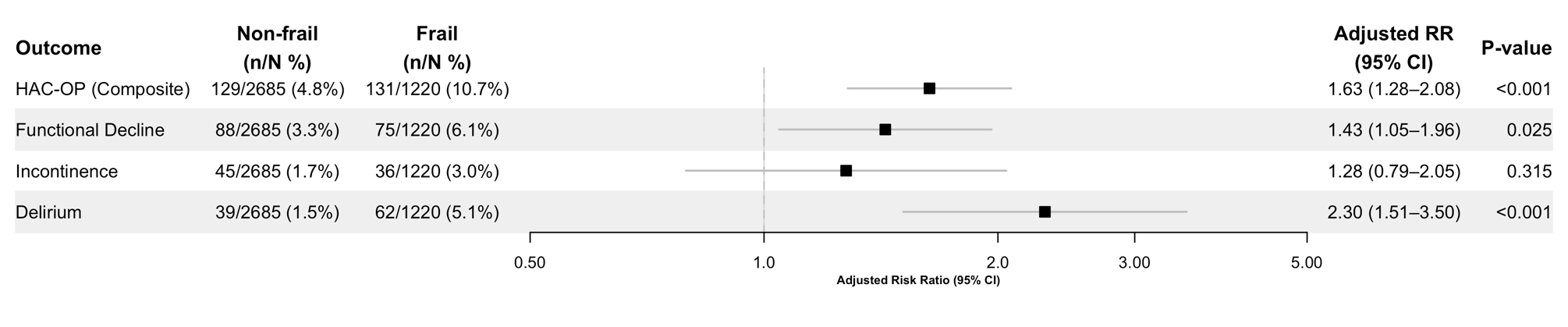


Because length of stay (LOS) may act as a mediator on the causal pathway from frailty to HAC-OP, LOS-adjusted estimates should be interpreted as direct effects. Models are adjusted for age (continuous), sex, body mass index, Charlson Comorbidity Index, Brinkman index, Barthel Index, IBD type, disease severity, time from first IBD diagnosis, hospital bed size, year of admission, and length of hospital stay.

Abbreviations: HAC-OP, hospital-associated complications of older people; RR, risk ratio; CI, confidence interval; LOS, length of stay.
